# Supplementary material for: Variations of organic functional chemistry in carbonaceous matter from the asteroid 162173 Ryugu
Source: Nat Commun. 2024 Aug 29;15:7488. doi: 10.1038/s41467-024-51731-w (PMC11362305; doi:10.1038/s41467-024-51731-w)
Supplement: Supplementary file 1 — Supplementary Information [file 41467_2024_51731_MOESM1_ESM.pdf]

Variations of Organic Functional Chemistry in Carbonaceous Matter from the Asteroid 162173 Ryugu

Bradley De Gregorio *et al.*

SUPPLEMENTARY INFORMATION:

Supplementary Figures 1-7

Supplementary Table 1

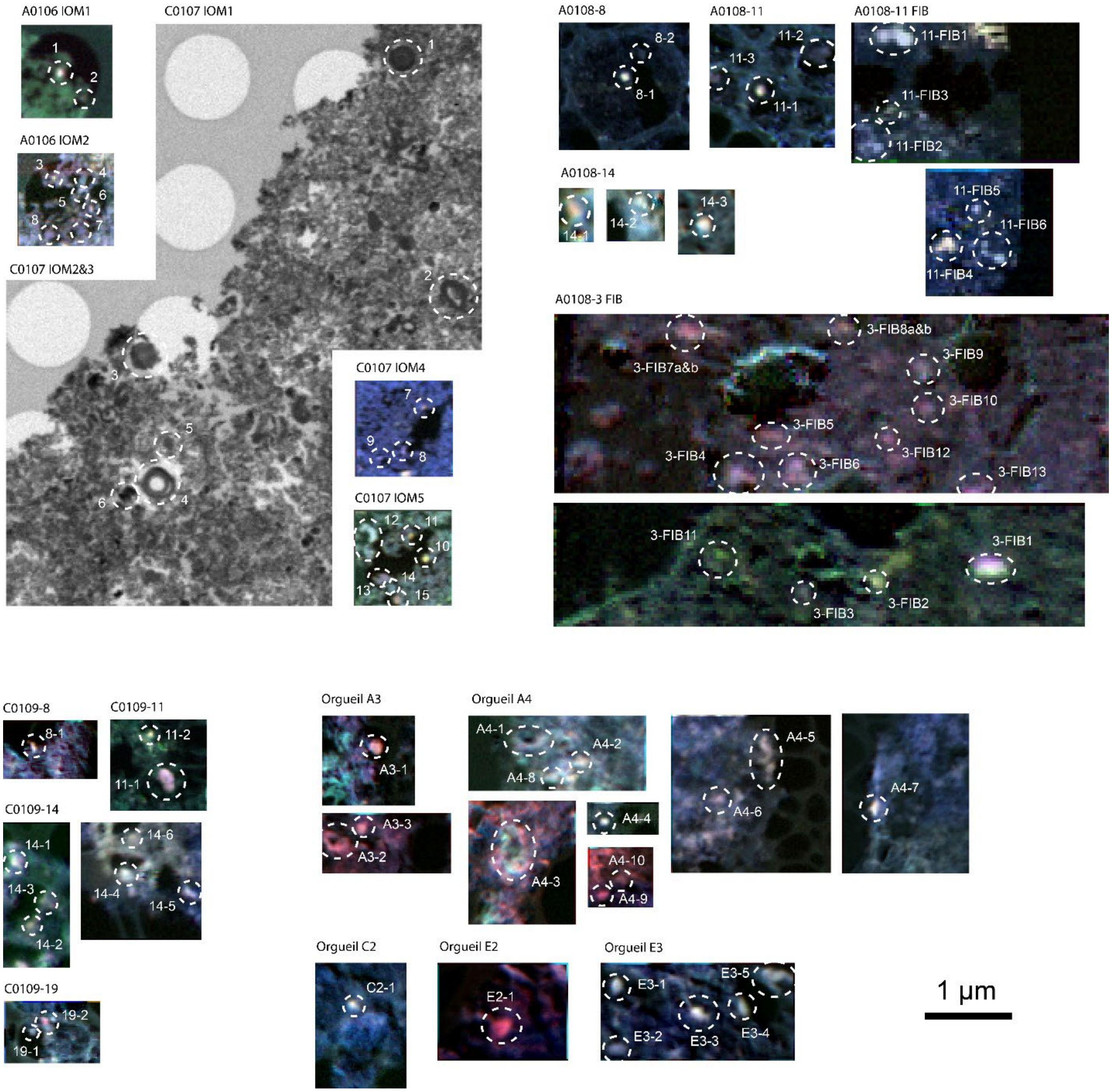

SUPPLEMENTARY FIGURE 1. Scanning transmission x-ray microscopy (STXM) images of Ryugu and Orgueil carbonaceous grains. Color figures combine three STXM images to show functional group variations (magenta = 285.0 eV; green = 286.7 eV; blue = 288.5 eV). All images are scaled to the same magnification.

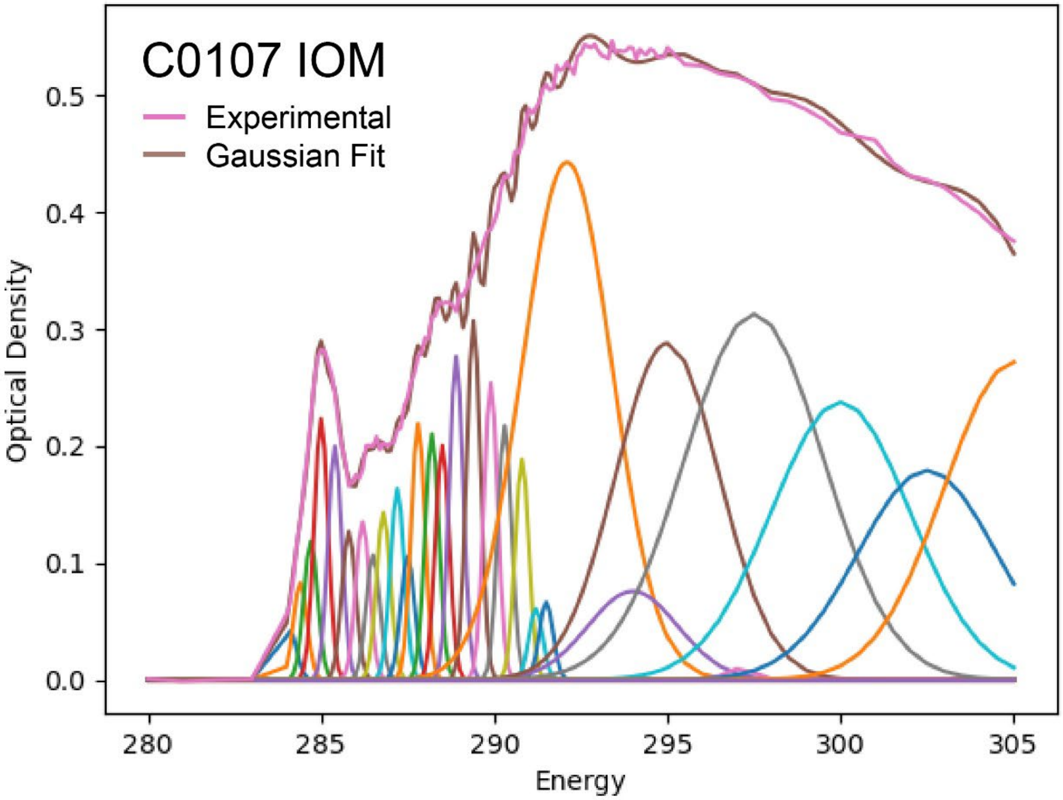

SUPPLEMENTARY FIGURE 2. Gaussian fitting result for Ryugu sample C0107 insoluble organic matter (IOM). The first 20 peaks have the same width, and their heights are used as parameters for hierarchical clustering to identify similar spectral shapes.

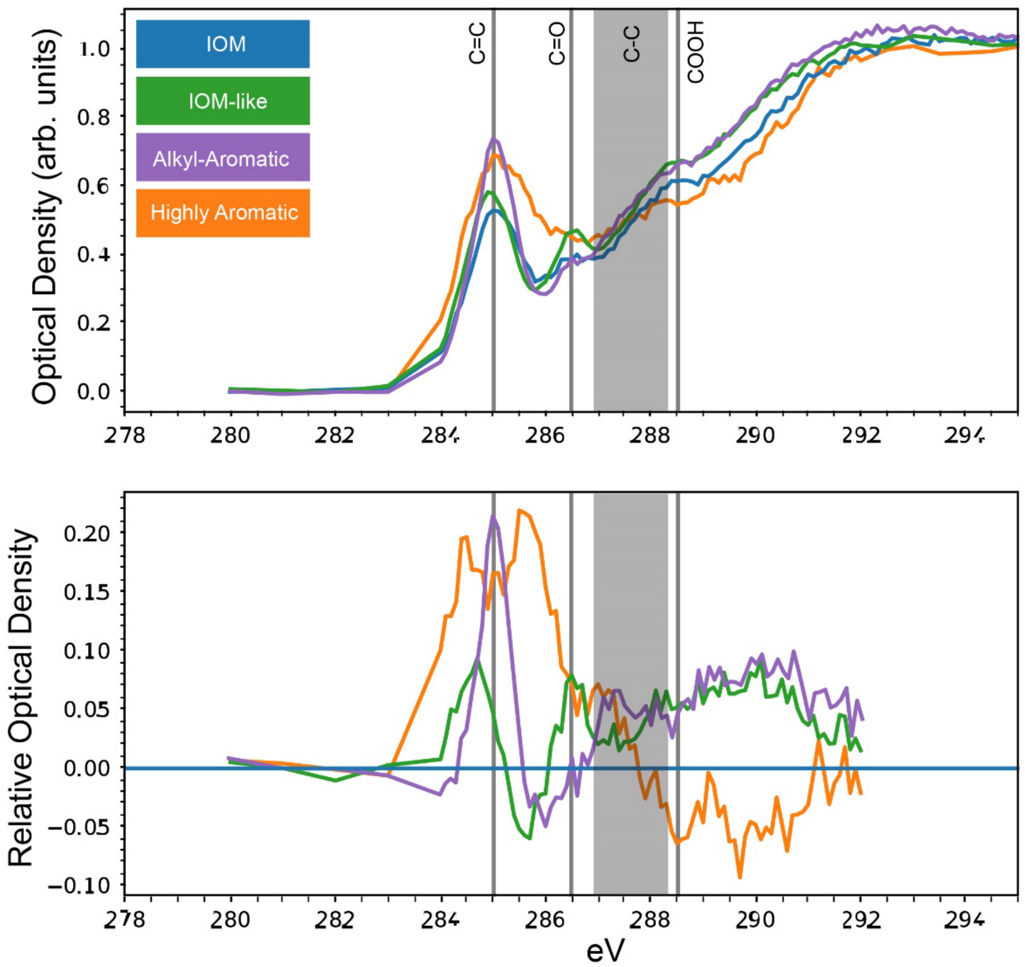

SUPPLEMENTARY FIGURE 3. Comparison of representative spectral shapes in insoluble organic matter (IOM) from sample C0107. The IOM-like (IL) shape is represented by C0107 grain 6 (B4 in Fig. 1). The Alkyl-Aromatic (AA) shape is represented by C0107 grain 1 (C3 in Fig. 1). The Highly Aromatic (HA) shape is represented by C0107 grain 10 (C1 in Fig. 1). The top plot shows the spectra normalized to edge height, while the bottom plot shows the spectra with the IOM spectrum subtracted. In the subtracted spectra, the broadening of the aromatic C=C peak at 285 eV for HA spectra, the extra ketone intensity at 286.5 eV of IL spectra, and the extra intensity between 287-289 eV for AA spectra can be seen.

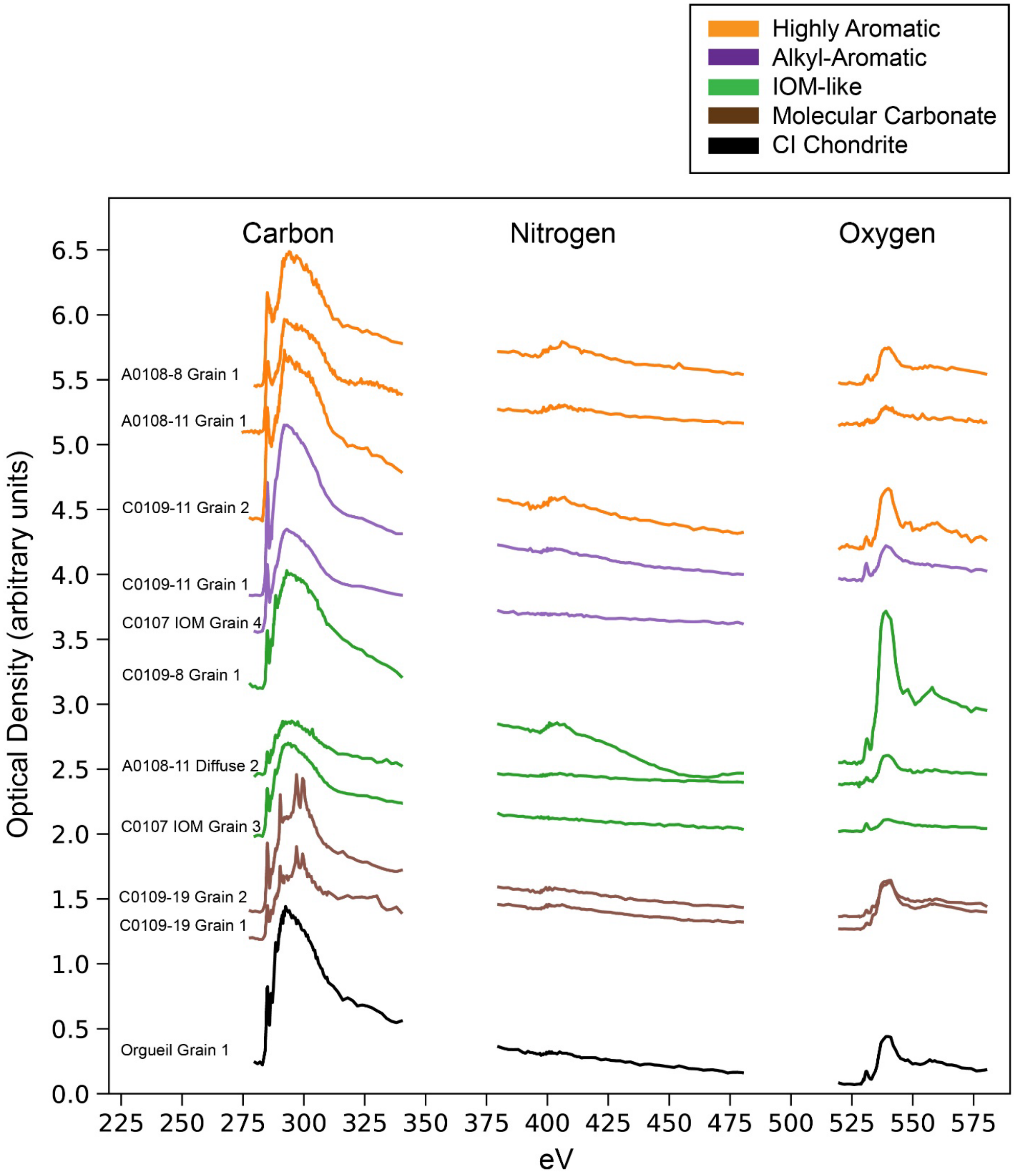

SUPPLEMENTARY FIGURE 4. Unmodified x-ray absorption near-edge structure (XANES) spectra of Ryugu and Orgueil carbonaceous grains spanning the carbon, nitrogen, and oxygen absorption edges. Spectra are color coded according to spectral shape, except for the grain from Orgueil in black. No oxygen spectrum was collected for C0107 insoluble organic matter (IOM) Grain 4. The dip in optical density seen in the nitrogen spectrum from C0109-8 Grain 1 is due to an unintended misalignment of focus during the latter portion of the scanning transmission x-ray microscopy (STXM) stack. The two grains from

C0109-19 that show the presence of molecular carbonate organic matter also include a pair of peaks due to potassium in the associated phyllosilicates. Care must be taken when interpreting the O-edge spectra, since a significant contribution to the spectra come from the surrounding silicate minerals.

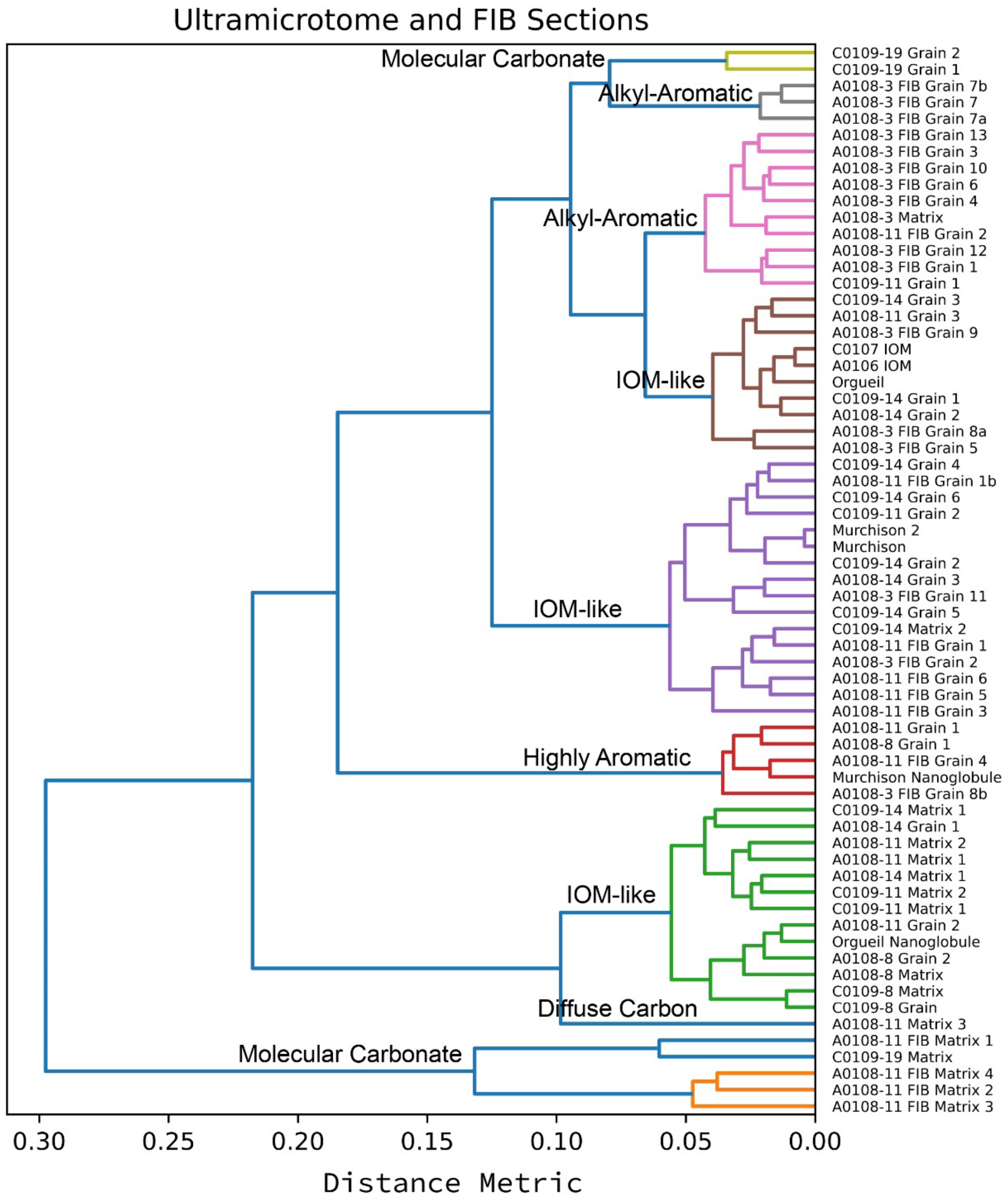

SUPPLEMENTARY FIGURE 5. Hierarchical clustering dendrogram of fitted x-ray absorption near-edge structure (XANES) spectra from Ryugu ultramicrotome and FIB liftout sections. The length of horizontal lines indicates the “distance” between spectral positions in a 21-dimensional space determined by Gaussian peak fitting. Samples labeled “Matrix” denote average spectra of diffuse carbon.

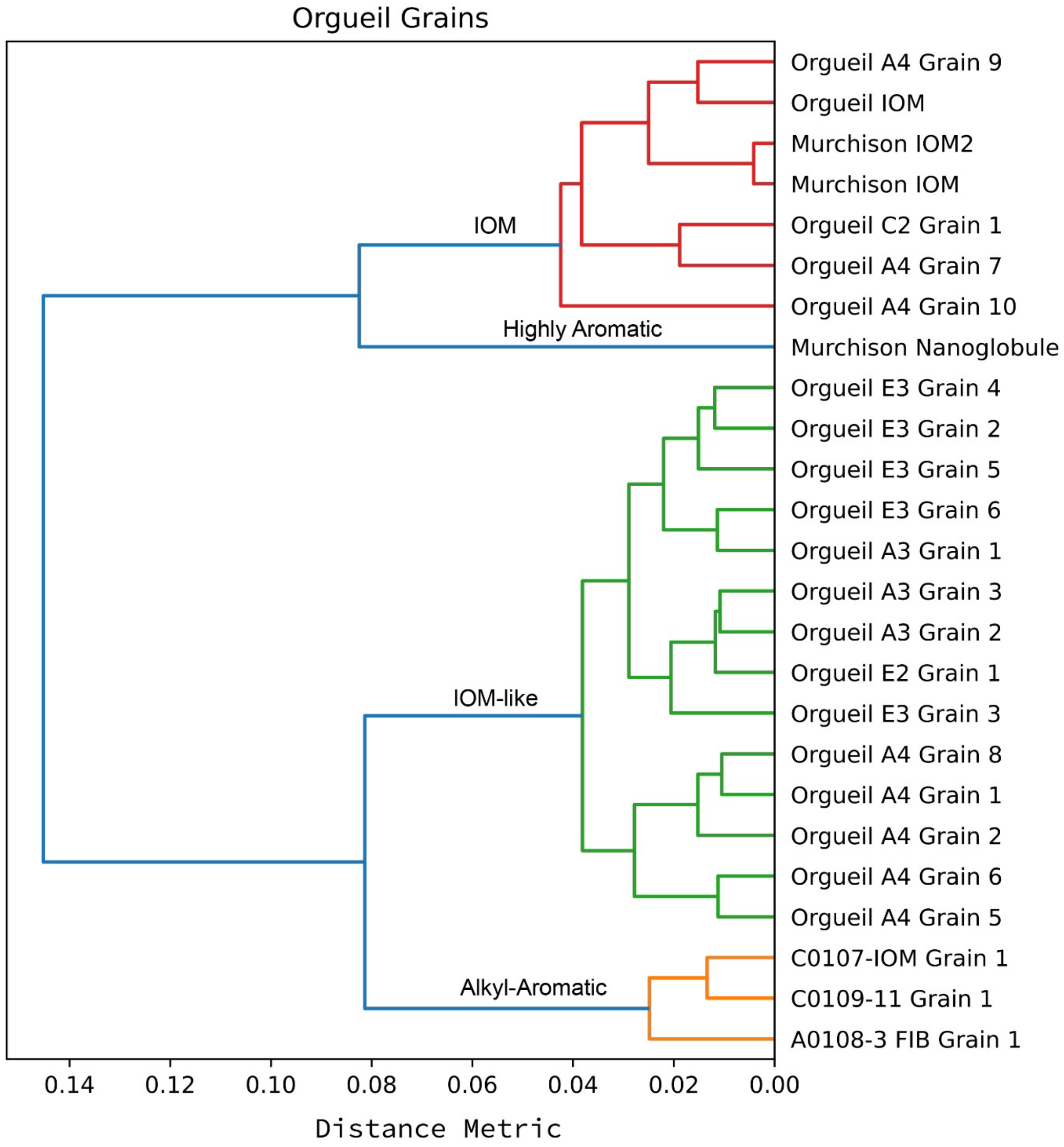

SUPPLEMENTARY FIGURE 6. Hierarchical clustering dendrogram of fitted x-ray absorption near-edge structure (XANES) spectra of carbonaceous grains from Orgueil ultramicrotome sections. The length of horizontal lines indicates the “distance” between spectral positions in a 21-dimensional space determined by Gaussian peak fitting. The “Murchison Nanoglobule” spectrum, representing Highly Aromatic (HA) XANES spectral shape, does not cluster with any other Orgueil grains. Similarly, the Alkyl-Aromatic (AA) spectra from Ryugu samples (shown in orange) cluster by themselves.

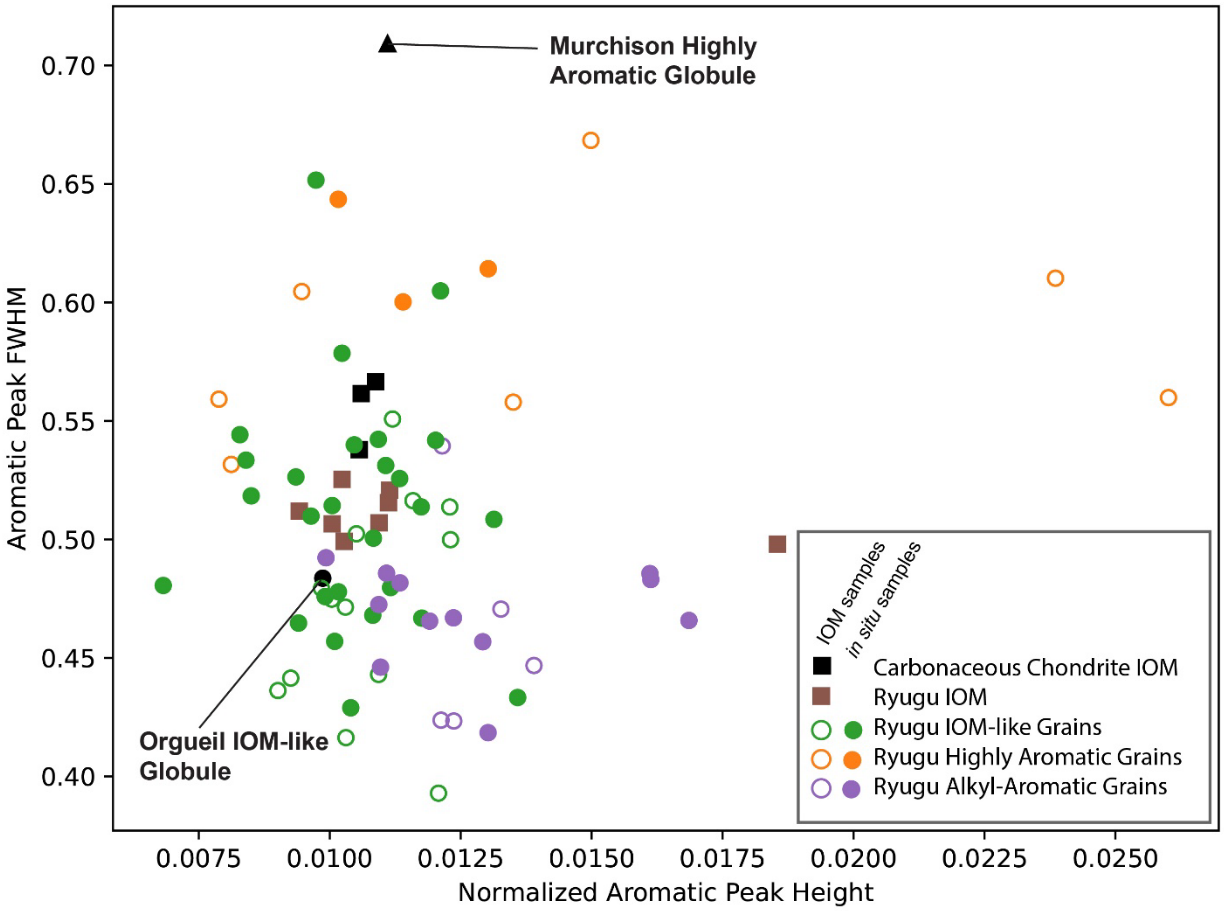

SUPPLEMENTARY FIGURE 7. X-ray absorption near-edge structure (XANES) spectral peak parameters for the aromatic (C=C) peak at 285 eV. Peak height and full-width half maximum (FWHM) values were estimated by fitting the aromatic and ketone peaks with two gaussians. The full spectral modeling used for XANES shape analysis uses four gaussians to capture the shape of the aromatic peak. This data shows that the Alkyl-Aromatic (AA) spectral shape has a sharp, narrow aromatic peak relative to most insoluble organic matter (IOM) and IOM-like (IL) spectra.

SUPPLEMENTARY TABLE 1. Isotopic Composition and Functional Chemistry of Carbonaceous Grains in A0108-3

**Grain**	**Previous Work[14]**	**XANES Spectral Shape[a]**	**$\delta^{15}N$**	**$\delta^{13}C$**	**CN/C_2**	**Number of σ from bulk $\delta^{15}N$**
1	3b	AA	-37 ± 53	-17 ± 15	0.51	1.659097
2		IL	70 ± 124	-57 ± 42	0.74	0.162754
3		AA	158 ± 122	-57 ± 38	0.68	0.885372
4	3c	IL	594 ± 60	-20 ± 15	0.63	9.025365
5	3a	HA	-89 ± 58	-73 ± 21	0.80	2.37146
6	3e	IL	235 ± 72	21 ± 22	0.67	2.567693
7a	3d	AA	234 ± 60	-1 ± 15	0.49	3.05445
7b	3d	AA	155 ± 65	31 ± 18	0.50	1.611534
8a		IL	407 ± 78	1 ± 28	1.15	4.609004
8b		HA	-120 ± 66	-35 ± 23	0.76	2.57429
9		IL	573 ± 53	5 ± 15	0.82	9.939644
10		AA	232 ± 95	14 ± 28	0.66	1.919197
11		IL	-35 ± 50	106 ± 25	1.39	1.708231
12		AA	232 ± 79	-37 ± 21	0.51	2.289107
13		AA	347 ± 123	-15 ± 32	0.57	2.407265
Bulk			53 ± 5	-17 ± 2	0.55	

[A]AA = Aromatic-Aliphatic; HA = Highly Aromatic; IL = IOM-like
